# Prevalence of Mental Distress Among Syrian Refugees With Residence Permission in Germany: A Registry-Based Study

**DOI:** 10.3389/fpsyt.2018.00393

**Published:** 2018-08-28

**Authors:** Ekaterini Georgiadou, Ali Zbidat, Gregor M. Schmitt, Yesim Erim

**Affiliations:** ^1^Department of Psychosomatic Medicine and Psychotherapy, Friedrich-Alexander University Erlangen-Nuernberg, Erlangen, Germany; ^2^Department of Psychiatry and Psychotherapy, Paracelsus Medical University, Nuremberg, Germany; ^3^Erlangen City Council, Job Center, Erlangen, Germany

**Keywords:** syrian refugees, PTSD, depression, generalized anxiety, post-migration variables, Germany, residence permission, mental health

## Abstract

**Background:** High rates of prevalence of mental distress among the Syrian refugee population have been repeatedly confirmed. However, little is known about the influence of length of stay, living conditions, and residence permission in the host country or about the duration of the escape journey and travel conditions on mental health in this refugee population. This study examines the mental health of Syrian refugees, taking into account the circumstances in their country of origin and host country, as well as their escape conditions.

**Methods:** This investigation formed part of a registry-based study. A sample of 518 adult Syrian refugees in Erlangen, Germany, who have residence permission was identified. The response rate was 38.6%; a total of 200 Syrian refugees thus participated in the study. The respondents were investigated for post-traumatic stress disorder (ETI), depression (PHQ-9), generalized anxiety (GAD-7) and post-migration variables.

**Results:** The prevalence of participants who had personally experienced and/or witnessed traumatic events was 75.3%. Symptoms of PTSD were found in 11.4% of the participants. Moderate to severe depression was confirmed in 14.5% and moderate to severe generalized anxiety in 13.5% of the sample. The criteria for at least one diagnosis were met by 30.5% of the participants. More severe PTSD symptoms were associated with older age, shorter validity of the residence permit, larger number of traumatic events (TEs) and higher generalized anxiety symptoms. Depression symptoms were associated with younger age, shorter duration of escape journey, larger number of TEs and higher generalized anxiety symptoms. Generalized anxiety symptoms correlated with female gender, PTSD, and depression symptoms.

**Conclusions:** These findings suggest that Syrian refugees in Germany are a vulnerable population, especially if they have experienced and/or witnessed multiple traumatic events. However, post-migration conditions and positive future prospects in the host country can be protective factors for this population.

## Introduction

It has been reported by the Office of the United Nations High Commissioner for Refugees that by the end of 2015, 65.3 million individuals were forcibly displaced worldwide. With 4.9 million refugees, 6.6 million internally displaced persons, and nearly 250,000 asylum seekers, an estimated 11.7 million Syrians were displaced by the end of 2015 and seeking protection within Syria or abroad (1). Since 2015, approximately 1.35 million asylum applications have been documented in Germany, including 473,881 requests from Syrians (2–4). Most Syrians receive political asylum—i.e., refugee status or subsidiary protection. In 2016, approximately 98% of the Syrians had received protection in Germany (5).

Coming from war regions, these people have often experienced traumatic events (TEs). This defines them as a high-risk group for mental disorders (6). Several studies have investigated the mental health problems of Syrian refugees in recent years. Prevalence rates range from 20.5 to 35.7% for PTSD (7–12), from 20 to 43.9% for depression (7–9, 12, 13), and from 19.3 to 31.8% for anxiety (8, 12).

Some of the risk factors for mental distress have previously been reported in the literature. Alpak et al. (10) found symptoms of PTSD in 33.5% of refugees from Syria in Turkey. They reported positive correlations between PTSD, the number of traumatic events experienced, and female gender. In a previous study by our group (8) on Arabic-speaking asylum seekers (34% of whom were Syrian) who had been placed in collective accommodation centers, with a mean length of stay in Germany of about 7.9 months, the prevalence of mental stress was high. Symptoms of PTSD were found in 35.7% of the sample, moderate to severe depression in 35.7%, and moderate to severe generalized anxiety in 39.3%. In comparison with asylum seekers who had mental distress, those without mental distress had experienced fewer TEs and had longer periods of stay in Germany. There were no differences between men and women with regard to the number of TEs, the prevalence of PTSD, depressive symptoms, or generalized anxiety symptoms (8). In a study that included internally displaced Syrians as well as Syrian refugees in the Netherlands, the rates of PTSD were 31.8% for internally displaced Syrians and 23.4% for Syrian refugees. However, the rate of depression was significantly higher in the refugee sample in comparison with the internally displaced Syrians (44.1 vs. 16%). The authors argue that internally displaced Syrians were continuing to live in the war zones and were therefore still in a conflict mode. An alternative explanation for the high depression rates among refugees is their sense of loss of their homeland, community, and family (14).

These results indicate the impact of gender and post-migration conditions on the prevalence of mental disorders. Variables affecting mental health, such as length of stay, living environment, and residence permission are rarely examined in research studies. Insecure residence status and poverty are relevant stressors in the acculturation process (15) and therefore have an impact on mental health (15, 16). Uncertainty about asylum status in the country of arrival gives rise to emotional distress (17), and the duration of the asylum procedure has been shown to be a risk factor for psychiatric problems (18). Bogic et al. (19) reported higher rates of PTSD among refugees with a temporary residence permit, while Chou (20) found that visa status is predictive for future psychological distress in older migrants. Traumatic events prior to migration, forced and unplanned migration, living alone or separated from family in the host country, and the length of migrant residence in the host country are known to increase the likelihood of mental disorders in migrants (21). In a study of refugees from Iraq, Nickerson et al. (22) found that post-migration living difficulties are a predictor for PTSD and depression. Steel et al. (23) assessed the long-term effects of trauma on mental health among Vietnamese refugees after a mean length of residence in Australia of 11.2 years. They reported that trauma-related mental illness appeared to decline steadily over time. They did not find any associations between several post-migration, social, and economic factors and mental illness. They suggest that post-migration stressors may decline after prolonged resettlement, but that the effects of high exposure to premigration trauma can persist.

When we examine the administrative process involved in obtaining asylum permission in Germany, several difficulties become apparent. Upon entry into Germany, refugees seeking asylum are required to formally request asylum. Verbally declaring “Asylum” to a local authority representative results in a change of the individual's status from illegal immigrant to asylum seeker. From their place of arrival, the refugees are allocated to different cities and regions. The initial distribution of asylum seekers is orientated in line with the “Königstein key”^1^. This key defines the percentage assigned to each federal state in matters affecting the national common financial adjustment scheme, such as the asylum system. It also determines what proportion of asylum seekers is to be received by each federal state. The distribution quota for 2016 in Bavaria was 15.5%. The asylum seekers can be accommodated in reception facilities (*Erstaufnahmeeinrichtung*) for up to 6 months, or until their application is decided on. If accommodation is provided in reception facilities, basic benefits are provided in the form of non-cash benefits (e.g., heating, clothing, personal hygiene, and benefits in case of sickness, pregnancy, and birth) and they are regulated by the Asylum Seekers' Benefits Act (*Asylbewerberleistungsgesetz*). Asylum applicants who are no longer obliged to live in a reception facility are distributed to follow-up facilities (for instance, collective accommodation or local accommodation, such as apartments or accommodation groups). The local Agency for Foreign Nationals mandates asylum applicants who have good prospects for remaining to attend language courses (*Integrationskurse*) whilst their applications are being processed. Asylum applicants from Eritrea, Iraq, Iran, Syria, and Somalia have been granted this early access because of the high likelihood that they will be granted asylum. At this stage, the applicants receive advice on the potential for limited access to the labor market. They can apply for counseling at the local employment agency and can look for work. Once they have received a job offer, they still need permission from the Agency for Foreign Nationals to start working. People who are granted asylum receive a temporary residence permit if the conditions for this are satisfied (§26 AsylG;^2^. In 2015, 105,620 decisions were made in Germany on asylum applications by Syrians (24). The vast majority of the applicants were granted refugee status in accordance with §3 Abs 1 AsylG^2^, following the Geneva Convention, which includes a 3-year resident permit.

Only 61 people were granted subsidiary protection status. This status implies a 1-year residence permit and may be extended for another 2 years. A major difference is the restricted subsequent immigration of dependants. People who were permitted to stay in Germany after March 16, 2016 on that legal basis are not permitted to bring members of their family to the country. This restriction is at present applicable until at least July 2018. The ratio for Syrians changed in the following years: of the 295,040 decisions made in 2016, 121,562 involved the granting of this subsidiary protection status within the meaning of Section 4 of the Asylum Act (*Asylgesetz*;^2^. In 2017, a majority consisting of 55,697 of the 99,527 decisions followed that legal basis. People with temporary residence permits and with subsidiary protection are allowed to work in Germany^3^. Necessary benefits are then no longer paid under the Asylum Seekers' Benefits Act, but unemployment benefits for jobseekers can be received under Book II of the Social Code (*Sozialgesetzbuch* II) if applied for. A single person is eligible to receive €416 per month in addition to the cost of housing. Attendance at an integration course is required in order to ensure good preparation for the labor market [§ 3 Abs 2a Satz 1 SGB II; (25)].

To the best of our knowledge, this is the first study on Syrian refugees in Germany who have residence permission. The aim was to examine mental distress among Syrian refugees who have been staying in Germany since 2014 and who have received a positive decision regarding their asylum procedure—i.e., a residence permit.

## Materials and methods

This investigation formed part of a registry-based study in Erlangen, Germany, and was conducted in collaboration with Erlangen City Council and the Job Center in Erlangen. Participants in the present study were Syrian refugees who arrived in Germany after 2014 and were resident in the city of Erlangen, receiving unemployment benefits and in possession of a residence permit. The registry of the Erlangen Job Center was used to obtain the data. At the time of the investigation, a total of 518 Syrian refugees were registered. All 518 Syrian refugees were informed about this study in written form. The purpose of the study and conditions for participation were explained to them. They were invited to participate and to attend prearranged meetings in a room at the city hall. Those who attended the meetings were again given information about the study orally. After consenting to participate, they were asked to complete questionnaires in Arabic. In the room, up to 15 refugees at one time completed the questionnaires during the meetings. The participants were asked not to speak to each other while filling in the questionnaires. The team members involved in the study (the first and second authors) and one translator remained in the room and offered further explanations if needed in German or Arabic.

The inclusion criteria for the study were: age of consent (minimum 18 years), agreement to participate in the study, Syria as the country of origin, and a good command of Arabic (at least spoken). Nineteen of the 200 participants (9.5%) were not literate, and the second author (AZ, Arabic-speaking, physician, doctoral candidate) read the questions for them and helped them to complete the questionnaires.

The data were obtained between July and December 2017. Participation in the study was completely voluntary, and written informed consent was obtained from all the participants. All of the study participants received a reimbursement of €15. The study was approved by the Ethics Committee of the University of Erlangen–Nuremberg (project identification code: 74_17 B).

### Assessment instruments

The survey instruments included sociodemographic and migration-related variables, as well as symptoms of post-traumatic stress, depressive symptoms, and symptoms of generalized anxiety disorder. The questions on sociodemographic and migration-related variables were translated from German into Arabic, back-translated, and commented upon by the first and last author. The comprehensibility and cultural validity of the Arabic versions of the questionnaires have been examined in a previous study (8).

#### Essen trauma inventory (ETI)

The Essen Trauma Inventory (26) is a self-rating questionnaire that was developed for assessing potential traumatic events and post-traumatic stress disorder. The translated version of the Essen Trauma Inventory was obtained from the author, Dr. Sefik Tagay. To begin with, the participants marked “yes” or “no” in the questionnaire if they had personally experienced or witnessed a series of potential TEs (e.g., torture, military conflict, explosions). For the worst TE that they had experienced, they rated whether it triggered an objective threat to life (A1 criterion) as well as a subjective feeling of threat (A2 criterion). Additionally, they rated the 17 items on the PTSD symptom list. The PTSD symptom list was rated on a 4-point Likert scale that ranged from “does not match at all” (0 points) to “completely matches” (3 points). Clinically apparent PTSD is indicated if the participant has experienced a TE, meets both criteria (A1 and A2), and the total score for the PTSD symptom list reaches a cut-off value of 27. Cronbach's α in the present study sample was α = 0.95.

#### Patient health questionnaire—depression module (PHQ-9)

Depression was assessed using the nine-item PHQ-9 (27, 28), which includes each of the nine DSM-IV (29) criteria for depression. The Arabic version of the PHQ-9 questionnaire is available online (30). The PHQ is a screening instrument for psychiatric case definition in primary care. The items refer to problems within the previous 2 weeks. The score for the severity of depression varied from 0 (not present at all) to 3 (present nearly every day), yielding a total score between 0 and 27. The scores for PHQ-9 were used to determine the presence of depression and its severity depending on the following score ranges: 10–14 moderate, 15–19 moderate to severe, and 20–27 severe. Cronbach's α in the present study sample was α = 0.70.

#### Generalized anxiety disorder (GAD-7)

An Arabic version (8) of the 7-item Generalized Anxiety Scale (31, 32) was administered in order to assess the symptomatic severity of generalized anxiety disorder among the participants. Each of the seven items was rated on a 4-point Likert scale. Response options were “not at all” = 0, “several days” = 1, “more than half the days” = 2, and “nearly every day” = 3. GAD-7 scores thus range from 0 to 21, with scores of ≥ 5, ≥ 10, and ≥ 15 representing mild, moderate, and severe symptom levels of generalized anxiety disorder. In this study's sample, the internal consistency was α = 0.92.

### Statistical analysis

Statistical analyses were conducted using the IBM SPSS statistical package, version 21.0 (IBM Corporation, Armonk, New York). Prevalence rates were calculated on the basis of available cut-off scores for each questionnaire. A missing value analysis was performed. In case of missing items, the value was replaced with the rounded mean of the remaining items in the corresponding questionnaire when maximally one item (ETI, GAD-7) and three items (PHQ-9) in the questionnaires were missing. In addition to descriptive methods, categorical variables including prevalence rates were compared using chi-square tests. Fisher's exact test was used if the expected count in one of the cells was less than 5. To calculate differences between groups, we used *t*-tests for continuous variables and chi-square tests for ordinal or dichotomous variables. If variables did not meet the assumption of normal distribution, the comparison between groups was repeated using nonparametric tests. After testing for multicollinearity, multiple linear regression analysis with the enter method was used to explore the influence of sociodemographic and migration-related variables on the severity of mental stress. The significance level for all tests was set at *p* = 0.050.

## Results

### Response rate and missing values

The response rate was 38.6%; a total sample of 200 participants came to the meetings and were included in the study. Analysis showed that the nonrespondents (*n* = 318) were significantly younger than those who participated in the study (29.6 years, *SD* = 9.9 vs. 33.3 years, *SD* = 10.6; *p* < 0.001) and were less frequently married (34.9% married among nonparticipants vs. 59.5% married among participants; *p* < 0.001), with no significant gender differences (25.2% women among nonparticipants vs. 30.5% women among participants, *p* = 0.183).

In the ETI questionnaire, eight participants (4%) had one missing item; in the PHQ-9 questionnaire, two participants (1%) had one missing item and 10 participants (5%) had three missing items; and in the GAD-7 questionnaire, eight participants (4%) had one missing item.

### Sociodemographic variables

The sample consisted of 61 women (30.5%) and 139 men (69.5%). The mean age of the participants was 33.3 years (*SD* = 10.6, range: 18–63). There were no significant age differences between women and men (*M* = 34.93, *SD* = 9.5 vs. *M* = 32.5, *SD* = 10.9, *p* = 0.071). Demographic data are presented in Table 1. A total of 114 participants (59.1%) were parents, and they had a mean of 3.4 children (range: 1–10).

**Table 1:** Sociodemographic variables.

**Characteristics**	**Women (*****n*** = **61)**		**Men (*****n*** = **139)**		**Comparison**		**Total (*****N*** = **200)**	
	***n***	**%^a^**	***n***	**%^a^**		***p***	***n***	**%^a^**
**MARITAL STATUS**								
Single	8	13.1	66	47.5	χ2 = 24.50	0.000	74	37.0
Married	48	78.7	71	51.1			119	59.5
Divorced/widowed	5	8.2	2	1.4			7	3.5
**AGE**								
18-24	11	18.0	40	28.8	χ2 = 8.41	0.089	51	25.5
25-34	19	31.1	42	30.2			61	30.5
35-44	19	31.1	39	28.1			58	29.0
45-54	11	18.0	10	7.2			21	10.5
>55	1	1.6	8	5.8			9	4.5
**ACCOMMODATION**								
Collective accommodation center	9	14.8	20	14.5	χ2 = 11.86	0.001	29	14.5
Own apartment (alone or with family)	49	80.3	83	60.1			132	66.0
Apartment together with other people	3	4.9	35	25.4			38	19.0
	**M (SD)**	**Range**	**M (SD)**	**Range**		***p***	**M (SD)**	**Range**
**EDUCATION IN YEARS**^b^								
(*n* = 186)	9.9 (4.9)	0−20	10.4 (4.3)	0−20	*t*_(184)_ = −0.617	0.538	10.2 (4.5)	0−20

a *Valid values*.

b *Including university attendance*.

### Psychiatric/psychotherapeutic treatment and migration-related variables

Twelve participants (6.0%) had received psychiatric/psychotherapeutic treatment at some time during their lives. Among them, five participants (41.7%) had treatment only in their country of origin, two participants (16.7%) had treatment in the country of origin and were receiving ongoing therapy in Germany and four participants (33.3%) were only receiving ongoing therapy in Germany. Migration-related variables are presented in Table 2.

**Table 2:** Migration-related variables.

**Migration-related variables**	**Women (*****n*** = **61)**		**Men (*****n*** = **139)**		**Comparison**		**Total (*****N*** = **200)**	
	***n***	**%^a^**	***n***	**%^a^**		***p***	***n***	**%^a^**
**ESCAPE JOURNEY**								
Across land and by boats/dinghies	43	71.7	119	85.6	χ2 = 12.9	0.002	162	81.4
Only across land	4	6.7	13	9.4			17	8.5
By another method (e.g., plane)	13	21.7	7	5.0			20	10.1
	**M (SD)**	**Range**	**M (SD)**	**Range**		***p***	**M (SD)**	**Range**
Escape duration^b^ (*n* = 161)	12.1 (30.2)	1–182	7.4 (17.6)	0.2–144	*U* = −0.004^f^	0.998	8.7 (21.8)	0.2–182
Escape difficulty^c^ (*n* = 193)	7.2 (3.3)	0–10	7.1 (2.9)	0–10	*U* = −0.660^g^	0.511	7.1 (3.0)	0–10
Duration of stay in Germany^d^ (*n* = 198)	21.1 (8.3)	2–50	24.3 (5.2)	10–52	*U* = −1.828^h^	0.068	23.3 (65)	2–52
Duration of residence permit^d^ (*n* = 178)	28.8 (13.3)	5–75	32.9 (10.3)	4–74	*U* = −2.357^i^	0.018	31.7 (11.3)	4–75
Future validity of permit^d^ (*n* = 180)	14.5 (8.8)	−9–32^e^	16.6 (8.5)	0–33	*U* = −1.394^j^	0.164	16.0 (8.6)	−9–33^e^
Duration of asylum procedure^d^ (*n* = 188)	8.8 (5.2)	1–20	9.3 (5.6)	0–27	*U* = −0.281^k^	0.780	9.2 (5.5)	0-27

a *Valid values*.

b *in weeks*.

c *visual scale from 0 (not at all difficult) to 10 (very difficult)*.

d *in months*.

e *negative values for current extension requests; mean ranks of women and men*.

f *81.02 and 80.99 respectively*.

g *101.04 and 95.31 respectively*.

h *88.28 and 104.38 respectively*.

i *75.57 and 94.94 respectively*.

j *81.90 and 93.90 respectively*.

k *92.75 and 95.21 respectively*.

The mean duration of their escape journeys was 8.7 weeks (*SD* = 21.8, range: 0.20–182). The majority (81.4%, *n* = 162) had traveled across land and by boats and rubber dinghies across the sea; 17 participants (8.5%) had traveled only across land; and 20 participants (10.1%) reported having reached Germany by another method (e.g., by plane). On a visual scale from 0 (not at all difficult) to 10 (very difficult), the difficulty of the escape was indicated as on average 7.1 (*SD* = 3.0, range: 0–10). Their mean duration of stay in Germany was 23.3 months (*SD* = 6.5, range: 2–52). Asylum procedures had lasted a mean of 9.2 months (*SD* = 5.5, range: 0–27). Their protection/residence permissions were valid for a mean of 16.0 months more (*SD* = 8.61, range: −9 to 33, with negative values for current extension requests). Residence permission had been issued for a mean of 31.7 months (*SD* = 11.3, range: 4–75).

### TES and post-traumatic stress disorder

A total of 149 participants (75.3%) had personally experienced and/or witnessed TEs; the distribution is shown in Table 3. Men had more often experienced and/or witnessed the following TEs: serious accident, fire, or explosion (χ^2^ = 8.31, df = 3, two-tailed Fisher's exact test *p* = 0.032) and physical violence from strangers (χ^2^ = 8.95, df = 3, two-tailed Fisher's exact test *p* = 0.023). Forty-nine participants (24.5%) reported only one TE, 76 participants (38.0%) reported two to five TEs, and 24 participants (12.0%) reported more than five TEs. Forty-six women (75.4%) and 103 men (75.2%) had experienced at least one TE (χ^2^ = 0.001, *df* = 1, two-tailed Fisher's exact test *p* = 1.000). On average, the participants reported having experienced and/or witnessed *M* = 2.27 TEs (*SD* = 2.39, range: 0–10). The most distressing TEs were military conflict (34.5%, *n* = 40), death of a loved one (17.4%, *n* = 20), and serious accident/explosion (13.0%, *n* = 15).

**Table 3:** Lifetime prevalence of traumatic events (*n* = 200).

**Traumatic event**	**Personally experienced**		**Witnessed**		**Personally experienced and witnessed**		**Personally experienced and/or witnessed**	
	***n***	**%^a^**	***n***	**%^a^**	***n***	**%^a^**	***n***	**%^a^**
Military conflict	62	31.2	20	10.1	3	1.5	85	42.7
Prisoner/hostage	21	10.7	22	11.2	1	0.5	44	22.3
Torture	12	6.0	4	2.0	2	1.0	18	9.0
Physical violence (stranger)	24	12.1	13	6.5	3	1.5	40	20.1
Physical violence (acquaintance)	6	3.0	5	2.5	2	1.0	13	6.6
Death of loved one (e.g., homicide)	37	18.5	18	9.0	4	2.0	59	29.5
Serious accident/explosion	38	19.4	26	13.3	5	2.6	69	35.2
Serious illness	10	5.1	29	14.8	0	0.0	39	19.9
Sexual harassment (stranger)	1	0.5	0	0.0	0	0.0	1	0.5
Sexual harassment (acquaintance)	3	1.5	0	0.0	0	0.0	3	0.5
Neglect	21	10.6	1	0.5	0	0.0	22	11.1
Childhood sexual abuse (stranger)	3	1.5	1	0.5	0	0.0	4	2.0
Childhood sexual abuse (acquaintance)	3	1.5	0	0.0	1	0.5	4	2.0
Natural catastrophe	7	3.5	6	3.0	0	0.0	13	6.5
Other trauma	32	16.4	8	4.1	0	0.0	40	20.5
At least one traumatic event	120	60.0	79	39.5	12	6.0	149	75.3

a *Valid values*.

Twenty-two participants (11.4%) met the criteria for a diagnosis of PTSD. There were no significant differences between women and men with regard to the prevalence of PTSD (16.4 vs. 8.6%, χ^2^ = 2.61, *df* = 1, *p* = 0.140) or the occurrence of PTSD symptoms [*M* = 37.2, *SD* = 4.4 vs. *M* = 34.5, *SD* = 5.8, *t*_(20)_ = 1.20, *p* = 0.243].

### Prevalence and severity of depression symptoms and symptoms of generalized anxiety disorder

The total score for depression in this sample was *M* = 7.1 (*SD* = 6.6, range: 0–27). There were no significant differences between women and men [*M* = 7.5, *SD* = 6.0 vs. *M* = 6.9, *SD* = 6.8, *t*_(198)_ = 0.63, *p* = 0.530]. The total score for generalized anxiety was *M* = 4.6 (*SD* = 4.9, range: 0–21). Women reported more generalized anxiety than men [*M* = 6.5, *SD* = 5.9 vs. *M* = 3.8, *SD* = 4.1, *t*_(198)_ = 3.3, *p* = 0.001]. The data for severity of depression and generalized anxiety in women and men are shown in Table 4.

**Table 4:** Severity of depressive symptoms and symptoms of generalized anxiety disorder.

**Measures**	**Women (*****n*** = **61)**		**Men (*****n*** = **139)**		**Comparison**		**Total (*****N*** = **200)**	
	***n***	**%**	***n***	**%**	**χ^2^**	***p***	***n***	**%**
**DEPRESSION (PHQ-9)**								
Total score 0–9	40	65.6	106	76.3	11.3	0.012	146	73.0
Total score 10–14	14	23.0	11	7.9			25	12.5
Total score 15–19	6	9.8	11	7.9			17	8.5
Total score >20	1	1.6	11	7.9			12	6.0
**GENERALIZED ANXIETY (GAD-7)**								
Total score 0–4	27	44.3	94	67.6	12.8	0.005	121	60.5
Total score 5–9	19	31.1	33	23.7			52	26.0
Total score 10–14	7	11.5	6	4.3			13	6.5
Total score >15	8	13.1	6	4.3			14	7.0

*PHQ-9, Patient Health Questionnaire—Depression Module; GAD-7, Generalized Anxiety Disorder*.

### Number of mental disorders

Sixty-one participants (30.5%) were screened as positive for at least one diagnosis. Thirty-three participants (16.5%) met the criteria in one diagnostic category, 14 participants (7.0%) screened as positive for two diagnostic categories, and 14 participants (7.0%) screened as positive for all three diagnostic categories. There were significant gender differences in the distribution of the number of diagnoses (χ^2^ = 8.5, *df* = 3, *p* = 0.043). Among the total sample, 139 participants (69.5%) did not have any mental distress. The distribution of the number of diagnoses of mental distress is shown in Table 5.

**Table 5:** Distribution of the number of diagnoses of mental disorders.

**Number of diagnoses**	**Women (*****n*** = **61)**		**Men (*****n*** = **139)**		**Comparison**		**Total (*****N*** = **200)**	
	***n***	**%**	***n***	**%**	**χ^2^**	***p***	***n***	**%**
Without diagnosis	38	62.3	101	72.7	2.1	0.182	139	69.5
**SINGLE DIAGNOSTIC CATEGORY**								
Only PTSD	1	1.6	4	2.9	0.4	1.000	5	2.5
Only depression	7	11.5	20	14.4			27	13.5
Only GAD	0	0.0	1	0.7			1	0.5
**TWO DIAGNOSTIC CATEGORIES**								
PTSD and depression	0	0.0	2	1.4	3.1	0.462	2	1.0
PTSD and GAD	1	1.6	0	0.0			1	0.5
Depression and GAD	6	9.8	5	3.6			11	5.5
**THREE DIAGNOSTIC CATEGORIES**								
PTSD, depression and GAD	8	13.1	6	4.3	5.0	0.035	14	7.0

*PTSD, post-traumatic stress disorder; depression, Patient Health Questionnaire-Depression Module total score ≥ 10; GAD, Generalized Anxiety Disorder total score ≥ 10*.

### Predictors of PTSD, depression, and generalized anxiety symptoms

Multiple linear regression analyses were performed to examine the influence of sociodemographic and migration-related variables on symptoms of PTSD, depression, and generalized anxiety (Table 6). Stronger symptoms of PTSD were significantly associated with older age (β = 0.24, *p* = 0.001), shorter future validity of residence permit (β = −0.20, *p* = 0.041), larger number of TEs (β = 0.35, *p* = 0.000), and greater symptoms of generalized anxiety (β = 0.46, *p* = 0.000).

**Table 6:** Linear regression analysis predicting symptoms of PTSD, depression and generalized anxiety (*n* = 200).

**Variable**	***R*^2^**	**Δ*R*^2^**	**B**	**SE B**	**β**	**95% CI**	***p***
**PTSD symptoms**	0.56	0.56					
Sex^a^			−1.08	1.53	−0.04	−4.10 to 1.91	0.481
Age			0.27	0.08	0.24	0.12 to 0.42	0.001
Years of education			0.09	0.17	0.03	−0.24 to 0.42	0.595
Being illiterate^b^			1.29	2.52	0.03	−3.68 to 6.26	0.610
Marital status^c^			−3.21	1.68	−0.13	−6.53 to−0.10	0.058
Accommodation^d^			0.24	1.34	0.01	−2.40 to 2.89	0.856
Escape journey^e^			−1.20	2.35	−0.03	−5.84 to 3.44	0.610
Escape duration			0.02	0.03	0.03	−0.04 to 0.08	0.527
Duration of stay in Germany			−0.16	0.14	−0.09	−0.43 to 0.11	0.244
Duration of residence permit			0.21	0.13	0.19	−0.04 to 0.46	0.093
Future validity of permit			−0.30	0.14	−0.20	−0.58 to−0.01	0.041
Number of TEs			1.77	0.32	0.35	1.13 to 2.40	0.000
PHQ-9 total score			0.08	0.15	0.05	−0.21 to 0.37	0.574
GAD total score			1.14	0.19	0.46	0.76 to 1.52	0.000
Ongoing psychiatric/psycho-therapeutic treatment			−0.01	0.01	−0.03	−0.02 to 0.01	0.563
**Depression symptoms**	0.63	0.63					
Sex^a^			1.46	0.76	0.10	−0.04 to 2.96	0.056
Age			−0.10	0.04	−0.16	−0.18 to−0.02	0.012
Years of education			−0.11	0.08	−0.07	−0.27 to 0.06	0.213
Being illiterate^b^			−1.72	1.26	−0.08	−4.20 to 0.76	0.173
Marital status^c^			1.09	0.85	0.08	−0.58 to 2.77	0.198
Accommodation^d^			0.37	0.67	0.03	−0.95 to 1.70	0.579
Escape journey^e^			−0.59	1.18	−0.03	−2.92 to 1.74	0.617
Escape duration			−0.04	0.02	−0.12	−0.07 to−0.01	0.012
Duration of stay in Germany			−0.03	0.07	−0.03	−0.16 to 0.11	0.716
Duration of residence permit			−0.09	0.06	−0.15	−0.22 to 0.03	0.140
Future validity of permit			0.10	0.07	0.12	−0.04 to 0.24	0.176
Number of TEs			0.67	0.17	0.25	0.35 to 1.00	0.000
GAD total score			0.82	0.09	0.61	−0.65 to 0.10	0.000
ETI symptom score			0.02	0.04	0.04	−0.05 to−0.09	0.574
Ongoing psychiatric/psycho-therapeutic treatment			−0.01	0.01	−0.06	−0.01 to 0.00	0.221
**GAD symptoms**	0.66	0.66					
Sex^a^			−2.06	0.52	−0.20	−3.08 to −1.03	0.000
Age			0.00	0.03	0.01	−0.05 to 0.06	0.931
Years of education			−0.04	0.06	−0.04	−0.16 to 0.08	0.467
Being illiterate^b^			−0.73	0.89	−0.04	−2.49 to 1.02	0.409
Marital status^c^			0.14	0.60	0.01	−1.05 to 1.32	0.824
Accommodation^d^			−0.10	0.47	−0.01	−1.04 to 0.82	0.814
Escape journey^e^			−0.04	0.83	−0.00	−1.68 to 1.60	0.963
Escape duration			0.01	0.01	0.06	−0.01 to 0.04	0.223
Duration of stay in Germany			0.06	0.05	0.08	−0.04 to 0.15	0.225
Duration of residence permit			−0.04	0.05	−0.08	−0.13 to 0.05	0.401
Future validity of permit			0.03	0.05	0.05	−0.07 to 0.13	0.561
Number of TEs			−0.07	0.12	−0.04	−0.31 to 0.17	0.543
ETI symptom score			0.14	0.02	0.35	0.10 to 0.19	0.000
PHQ-9 total score			0.41	0.04	0.55	0.32 to 0.49	0.000
Ongoing psychiatric/psycho-therapeutic treatment			0.00	0.00	0.02	−0.01 to 0.01	0.602

a *0 = female, 1 = male*;

b *0 = no, 1 = yes*;

c * 0 = unmarried, 1 = married*;

d *0 = collective accommodation center/ with others, 1 = own apartment or with family*;

e *0 = mainland incl. sea, 1 = other*.

*ETI, Essen Trauma Inventory; GAD, Generalized Anxiety Disorder, PHQ-9, Patient Health Questionnaire—Depression Module; PTSD, post-traumatic stress disorder; TE, traumatic event*.

More severe symptoms of depression were significantly associated with younger age (β = −0.16, *p* = 0.012), shorter escape journey duration (β = −0.12, *p* = 0.012), larger number of TEs (β = 0.25, *p* = 0.000), and greater symptoms of generalized anxiety (β = 0.61, *p* = 0.000).

Increased symptoms of generalized anxiety were significantly associated with female gender (β = −0.20, *p* = 0.000), more severe PTSD symptoms (β = 0.35, *p* = 0.000), and stronger depression symptoms (β = 0.55, *p* = 0.000).

## Discussion

The present study examined the prevalence of TEs and the occurrence of symptoms of PTSD, depression, and generalized anxiety in Syrian refugees who have been living in Germany since 2014 and have residence permits. The main finding of the present study is that lower levels of PTSD, depression, and generalized anxiety were observed in comparison with other studies on Syrian refugees. We would attribute this result to the optimized living conditions in the sample, emphasizing the importance of post-migration variables.

Approximately 11.4% of the participants in the study met the criteria for PTSD, while 14.5% met the criteria for at least moderate depression and 13.5% met the criteria for moderate generalized anxiety. Approximately one-third of the participants met the criteria for at least one diagnosis. These results are in contrast to the findings of previous studies from different countries that have shown high prevalence rates of mental distress among Syrian refugees (7–13).

However, post-migration conditions and visa status have not been taken into account in the majority of studies on refugees. Richter et al. (9) examined mental distress among refugees in reception facilities. The participants were still going through the asylum procedure at that time, with a short length of stay in Germany. Other studies have also focused on asylum seekers (2, 10, 13). Kazour et al. (11) included Syrian refugees living in camps in Lebanon, with a mean duration of displacement of 10 months. In a previous study by our group on Arabic-speaking asylum seekers who had been placed in collective accommodation centers (8), the participants did not have residence permission and their mean length of stay in Germany was relatively short (7.9 months). However, a negative correlation was found between the severity of mental distress symptoms and the length of stay in Germany.

Longer duration of the asylum procedure has been shown to be an important risk factor for psychiatric problems (18). In the context of the acculturation process, an unclear residence permission status and poor economic conditions (and living conditions) are stress factors for immigrants (15). Silove et al. (17) reported that delays in processing refugee applications can increase the refugees' stress in the country of arrival. In refugees from the former Yugoslavia, Bogic et al. (19) found that temporary residence permission status was associated with higher rates of mood and anxiety disorders. Studies on refugees from Iraq (22), from Syria (12), as well as Arab immigrant women (33) also confirm the importance of post-migration conditions on mental health. By contrast, Steel et al. (23) did not find any association between several post-migration, social, and economic factors and mental illness among Vietnamese refugees.

Different visa status among the participants in the present study in comparison with other studies on Syrian refugees might explain the wide divergence noted in the prevalence rates of mental distress. In addition to residence permission, general living conditions in the host country are also important factors for mental health. Even in EU member states in which asylum policies are similar, once the refugees have obtained residence permission their living conditions vary from country to country, due to differences in the national welfare systems (34). In Germany financial support for every child is approximately €194–225 per month. In Greece, for example, there are no benefits for families, and some support is available only for those with many children and a low income. In the case of illness or inability to work, Germany provides financial support relative to the last income before the illness. In Greece, there is no such benefit (35). Additionally, the unemployment rates in these countries differ [approximately 5.7% in Germany vs. 22.3% in Greece in 2017; (36, 37)]. Psychotherapeutic care for refugees also differs very widely between EU member states. Although countries may legally be obliged to provide equal treatment to asylum seekers and citizens, refugees may still not be entitled to outpatient psychotherapy treatment if their health insurance does not cover it^4^. In summary, it can be seen that social welfare systems and conditions for refugees differ in the EU member states and that the benefits in some countries are much higher than in others. Prospects for obtaining residence permission as a refugee also differ between countries. For Greece, data from 2016 show that 70.9% of applications from asylum seekers from all countries are rejected, whereas in Germany only 28.6% of applications are rejected^5^. For refugees, it is crucial to know that after all of their difficulties and problems (e.g., escaping from their home country and experiencing multiple traumatic events, sometimes even during the escape), they will be able to stay and receive protection in a country that also provides good prospects for their future and for a new start in life. These could be factors that can help this vulnerable group on their way to mental stability.

However, in a large study of mental health among Syrian refugees resettled in a European country (Sweden) in which the national welfare system is comparable to that in Germany, the prevalence rate of mental disorders is reported to be in the range of 30–40%, and approximately 55% of the refugees had at least one mental disorder (12). Despite the comparability of Germany and Sweden with regard to residence status, length of stay in the host country, and general living conditions, the present study found lower prevalence rates of PTSD, depression, and anxiety in Germany. However, this discrepancy might be attributed to significantly larger numbers of traumatic events experienced by the individuals in the Swedish sample. In the study by Tinghög et al. (12), the refugees had experienced a mean of 4.2 traumatic events, while in the present study the participants reported having experienced and/or witnessed 2.3 traumatic events. In addition, the types of traumatic event experienced differ between the sample in Sweden (e.g., military conflict 86.9%, torture 30.6%, physical violence or assault 30.5%, witnessing physical violence or assault 63.3%, sexual violence 6.9%) and the sample in Germany (e.g., military conflict 42.7%, torture 9.0%, personally experienced and/or witnessed physical violence from a stranger 20.1%, sexual violence from a stranger 0.5%). Another possible explanation for differences in the samples might be different recruiting methods (postal questionnaire versus personal appointment). It might be presumed that individuals who participate through a personal appointment are those with greater emotional stability, as they have to organize attendance. These differences emphasize the fact that refugees are generally a heterogeneous population with different premigration and post-migration conditions that depend on the country in which they arrive. The symptom manifestations measured may also depend on methodological aspects such as broader inclusion criteria.

Studies on refugees also need to take into account comparisons with the population in the countries of origin. Interestingly, the present results show prevalence rates of mental illness similar to those in the Arabic population in general. The review by Tanios et al. (38) reported different rates of anxiety disorders in Arab populations, at 28.2% in Jordan, 16% in Saudi Arabia, 16.7% in Lebanon, and 10% in the United Arab Emirates. In Lebanon, Karam et al. (39) noted a 3.4% lifetime prevalence rate of PTSD, a 16.7% prevalence rate of anxiety, and a 12.6% prevalence rate of mood disorders. The lifetime prevalence of any disorder was 25.8%. However, in a study in four post-conflict areas, the lifetime prevalence for PTSD in the Gaza Strip was 17.8% (40).

In studies with participants from the Middle East, an association is seen between mental disorders and various socioeconomic variables (10, 41–48) and also traumatic events (10, 41, 42, 44, 47). In the present study women had a significantly higher severity of depression and generalized anxiety than men and female gender was a significant predictor for GAD symptoms. In addition to socioeconomic variables and the number of TEs, the present study found an association between reported PTSD and a shorter future validity of residence permits. The shorter future validity may be interpreted by the participants as representing uncertainty about their further stay in the host country and may give rise to fear about returning to their homeland. The present study found associations between higher depression symptom scores and a shorter escape duration. A possible explanation for this could be that people with shorter escape durations had had less time to mourn and to adapt to the fact of leaving their homeland.

The strengths of the present study include its registry-based methodology, the sample size representing the largest refugee population reported in Germany since 2014, the inclusion of information about living conditions, and the good comparability of the demographic characteristics of the sample with the basic Syrian refugee population in Germany. In the period from 2014 to 2017, 64.8% of requests for asylum from Syrian refugees were made by men and 35.2% by women (2–4, 49). In the present sample, 30.5% of the participants were women and 69.5% were men. Among applicants in Germany between 2014 and 2017, 22.6% were aged 18–24 and 34.7% were aged 25–44 (2–4, 49). In the present survey, 25.5% of participants were 18–24 years old and 29% were 25–44 years old. The mean duration of the asylum procedure in 2016 was 8.7 months (3). In the present sample, the asylum procedure had lasted a mean of 9.2 months.

Despite these strengths, the study also has some limitations. First of all, the response rate represented 38.6% of the registry-based total sample. A possible reason for the low response rate could be the recruiting method (personal prearranged appointments that may have collided with other appointments such as language courses, medical appointments). However, it was noted that responders were older and were more often married than nonresponders, and this might also represent a selection bias, with nonresponders having higher mental distress, based on the results showing that age is a predictor for PTSD and depression. An additional shortcoming of the investigation is the lack of information on psychiatric comorbidities other than PTSD, depression, and generalized anxiety; the self-reported questionnaires could also be a source of bias. In addition, the cross-sectional design of the study does not make it possible to draw any causal conclusions concerning the influence of the variables measured.

## Conclusion

These findings suggest that Syrian refugees in Germany are a vulnerable population, particularly if they have experienced and/or witnessed multiple traumatic events. The survey cohort was not in distress to the same extent as previous studies with this population in Germany have shown. Post-migration conditions and good prospects in the host country appear to be a protective factor after refugees have escaped. It can also be presumed that refugees first focus on integrating into the country of arrival and that mental distress increases over time.

## Recommendations for future research

Further research and prospective studies with representative samples of refugee populations are needed in order to confirm and refine these results. The present survey has shown the difficulty of comparing results, since asylum policies and living conditions in the resettlement destinations are different. Future studies should therefore assess not only premigration factors (e.g., traumatic events) but also different post-migration conditions (e.g., residence status, living conditions, acculturation, escape conditions) that can become sources of stress. It is also important to identify protective factors (e.g., social support). Since traumatic events of this intensity and severity can be transmitted intergenerationally, future studies with this population should also include their children. Germany and Europe have been witnessing the largest migration recorded in their history in recent years, and the provision of care for refugees has become a challenge for the mental health systems in the host countries. Establishing an EU database that gathers together academic research studies from the different member states on the topic of refugees and mental health might be helpful in efforts to understand and plan the corresponding requirements in the health services.

## Data availability

The raw data supporting the conclusions of this study will be made available by the authors upon request.

## Author contributions

EG conceived, designed, conducted, analyzed and wrote this manuscript. AZ and GS contributed to manuscript writing. YE provided feedback and mentorship at each stage of the research design and implementation, including a full review and provision of feedback on the final manuscript.

### Conflict of interest statement

The authors declare that the research was conducted in the absence of any commercial or financial relationships that could be construed as a potential conflict of interest.

## Footnotes

^1^Bundesamt für Migration und Flüchtlinge. Available online at: http://www.bamf.de/EN/Fluechtlingsschutz/Ankunftszentren/ankunftszentren-node.html (2017).

^2^Bundesministerium der Justiz und für Verbraucherschutz. Asylgesetz (AsylG) Available online at: http://www.gesetze-im-internet.de/asylvfg_1992/AsylG.pdf (1992).

^3^Die Bundesregierung. Available online at: https://www.bundesregierung.de/Content/DE/Lexikon/FAQ-Fluechtlings-Asylpolitik/2-was-bekommen-fluechtlinge/03b-Leistungen-Anerkannte.html (2018).

^4^Bundespsychotherapeutenkammer. *Psychotherapeutic Care for Refugees in Europe*. Available online at: http://www.npce.eu/mediapool/113/1137650/data/20171006/psychotherapeutic_care_for_refugees_in_europe.pdf

^5^Asylum Information Database. Available online at: http://www.asylumineurope.org/
